# Notes and News

**Published:** 1890-03-08

**Authors:** 


### Notes and News.

Collected and Communicated.

The Mayor of Durham (Mr. Richardson Peele) has handed over to the treasurer of the Durham County Hospital the sum of £88, being the net proceeds of the fancy and evening dress ball held in the Town Hall, Durham, on January 22nd, in aid of the hospital funds.

The Great Northern Central Hospital treated 861 in-patients and over 15,000 out-patients last year, the total ordinary expenditure being £5,764. A coffee-stall has been started for the out-patients, and the Hospitals Association has kindly supplied an ambulance litter for use in the district. A house has also been taken for the nurses, and the rooms thus freed are used for delirious and insane patients. Large though it is, this hospital is not adequate for the neighbourhood, and the committee look forward eagerly to the completion of the building.

The Abernethian Society held a conversazione at St. Bartholomew's Hospital on February 25th, when students and nurses were in great force. The library, large theatre, and physiological laboratory were all open to the guests, and some interesting exhibits were on view. Perhaps the head of a mummy cat and the spirometer attracted most attention. The phonograph talked to a large audience, and there was some good music. Miss Barling stood up in uniform and sang "Last Night," and of course secured loud applause from her comrades. Altogether, the society is to be congratulated on the success of their entertainment.

On October 27th was opened the new Kendray Fever Hospital at Barnsley, the Mayor, Dr. Sadler, Dr. Whitelegge and many other dwellers in the neighbourhood being present. The hospital is a well-built structure, situate in a commanding position on the top of Measborough Hill, a short distance from the town, and consists of three blocks—the administrative block, the isolation block, and the small-pox block. These are each distinct and apart from the others, and are fitted with every appliance. There is accommodation for about twenty patients. The hospital was erected from plans of Messrs. Morley and Woodhouse, of Barnsley, at a cost of £5,000, given by Mrs. Lambert, the site being provided by the Corporation at a cost of £2,300. The hospital is named after Mrs. Lambert's father.

The Convalescent Fund of St. Mary's Hospital is applying for help. A few shillings from each annual subscriber to the hospital would place the fund in a flourishing condition, so surely the appeal will not be in vain. A hospital without a convalescent fund is but a poor crippled institution, for patients cured of some fell disease or injury are yet often so weak from the after effects that, without a rest by the sea, they may in many cases remain invalids for life.

A horrible series of crimes is reported to have been discovered at Warsaw. A fire at an old house in Sienna Street had been extinguished by the brigade, when some firemen discovered a child's corpse beneath the floor; two other similar discoveries were made afterwards, and finally evidence was discovered of over 100 infanticides. An inmate of the house, a midwife named Skoblinska, has been arrested on suspicion of having killed and buried the infants.

The Middlesex Hospital issues, as usual, a very full and very satisfactory report. The total number of patients treated last year exceeded 3,000, the expenditure exceeding £28,000. The chapel and new buildings, from designs generously given by Mr. Keith Young, were finished in November last. Once more we beg our readers to remember the cancer wards of this hospital, and to subscribe to their support. The festival dinner will take place on March 21st, the Duke of Fife in the chair. A splendid list of stewards is already issued.

Hunts County Hospital treats a steadily-growing increase of patients, the total number of persons who took advantage of the hospital last year being 2,417. Luckily the subscribers are also increasing, and the hon. secretary announces with pardonable pride some 27 new annual subscriptions. Quite a different tale is told of Stanley Hospital, Liverpool, where 40 beds cannot be used for want of funds, and the subscription list is only £500. Newark Hospital again issues a flourishing report, showing an increase of patients, and a further investment of funds. We confess to a preference for those hospitals which are not proud of being in debt.

The late Mr. John Fleming, of Newcastle-on-Tyne, who died recently, has bequeathed some very large sums to various local charities. Mr. Fleming was a well-known philanthropist, and during his lifetime had erected the Fleming Memorial Hospital for sick children. To this institution he bequeaths £25,000; to the Newcastle Infirmary is left the sum of £10,000; the Prudhoe Convalescent Home at Whitley, and the Village Home for Girls at the same place, each get £2,500. Twelve institutions receive £500 apiece, three £300, one £250, seven £200, three £150, and one £100. The total amount of charitable bequests exceeds £100,000.

At the annual meeting of the Leeds Infirmary, the Chairman, Mr. R. Benson Jowitt, in moving the adoption of the report, said that the past year, like all others they had lately had to chronicle, showed a large increase. The increase had been all round—in work largely, in income considerably, in expenses to only a small extent, and that, he thought, was satisfactory. There had been five beds more occupied all the year round, and including those at the Ida Hospital, which was really a ward of that infirmary, there had been 5,737 in-patients during the year. The plans for the extension of the infirmary are now in a forward state, and the building fund, thanks to the treasurer's energy, is growing satisfactorily. The departure of Miss L. M. Gordon to a larger sphere of work was commented on in the following flattering terms: "They congratulated Miss Gordon, who had shown while with them great administrative capacity, uniform kindness and geniality, combined with firmness and absolute absence of fussiness, she being always tranquil, calm, and pleasant to act with."

THE following vacancies are announced this week, the amount of the salary in each case being given after the name of the institution:

**Resident Medical Officers.**

Addenbrooke's Hospital, House Surgeon—£65, board, etc.

Alnwick Infirmary, House Surgeon—£120, board, etc.

West London Hospital, House Surgeon—Board, etc.

West Riding Asylum, Pathologist—£100, board, etc.

Royal Free Hospital, Junior Medical Officer—Board, etc.

Southport Infirmary, House Surgeon—£100, board, etc.

Wallasey Dispensary, Junior House Surgeon—£80, rooms, etc.

**Non-Resident Medical Officers.**

County Council of Lancaster, Medical Officer of Health—£800.

County Council of Worcester, Medical Officer of Health—£600.

Durness, Sutherland, Medical Officer—£150.

Farringdon Dispensary, Honorary Surgeon.

King's College, Professor of Physiology.

London Hospital Medical College, Two Demonstrators, £90.

Owen's College, Manchester, Lecturer on Diseases of the Larynx.

Muthill, Perthshire, Medical Officer—£40.

Hospital for Women, Soho, Clinical Assistants.

---

THE Quarterly Court of Governors of the Hospital for Consumption at Brompton was held last week. The report of the Committee of Management, read by the secretary (Mr. Dobbin) stated that since the last court the whole of the 321 beds in the two buildings had been continuously occupied. Of the number of in-patients benefited, 72 had had the further advantage of some weeks' residence in convalescent homes on the south coast, their entire expenses having been defrayed by the hospital. Funds are constantly needed to enable the committee to carry on the great and useful work of the charity.

---

ON February 26th, Mr. Beamish formally opened the new workhouse infirmary for Coventry, and in the evening entertained about 100 gentlemen at dinner to celebrate the occasion. All the improvements which modern sanitary science offers have been taken advantage of in the building, and leave nothing to be desired; and the building is fitted throughout with pneumatic bells. In addition to the Manchester grates in the wards, the infirmary is heated by a system which supplies hot water for domestic and bathing purposes and for the coils in the corridor and some of the rooms. Messrs. Steane, of Coventry, are the architects, and the infirmary from the road presents a length of 230 ft.; the building material being red bricks interlaced with some bands of blue brick and Bath stone. The effect will be prettier when mellowed by age.

---

AMONG those present at the meeting of the Hospitals Association on February 26, at the Charing Cross Hospital, were: Mr. E. Murray Ind (Chairman of the London Hospital), in the chair; Mr. John C. Brasier (National Hospital for Paralyzed and Epileptic), Mr. T. Fowell Buxton, Mr. T. Beattie Campbell (Secretary, Royal Westminster Ophthalmic Hospital), Miss A. Glanfield Clark, Mrs. Franks, Mr. H. A. Gifford (Secretary, London Skin Hospital), Miss Flora Goldsmid, Miss Gordon, Mr. H. Hargrave Graham (Secretary, Hospital for Epilepsy), Mr. A. S. Hincks (Secretary, Gordon Hospital for Fistula), Mr. G. Q. Roberts (Secretary of the London Hospital), Dr. Howell, Mr. G. B. Lloyd, J.P. (General Hospital, Birmingham), Mr. J. Michelli, Lieut.-Colonel Montefiore, Sir William Moore, K.C.I.E., Mr. W. J. Nixon (House Governor, London Hospital), Mr. S. M. Quennell (Secretary, Westminster Hospital), Mr. Arthur E. Reade (Secretary, Charing Cross Hospital), Mr. G. Owen Ryan (Secretary, Queen Charlotte's Hospital), Mr. Thomas Ryan (Secretary, St. Mary's Hospital), and Mr. Conrad W. Thies (Secretary, Royal Free Hospital). A discussion followed the reading of Mr. Burdett's paper, which will be found in another column, in which Mr. Murray Ind, Mr. Nixon, Mr. Quennell, Mr. Thomas Lloyd, J.P., and Mr. Hargrave Graham took part.

WE have received number four, being the monthly issue for January, of the *State Charities Record*, the authorised periodical of the State Charities Aid Association of New York. It contains much interesting matter, and we welcome this important departure on the part of one of the most useful and beneficial associations in the whole world. We would suggest the addition of a list of contents, and also a column recording, from time to time, changes in the staff in the various charities in which the association is interested. The *Record* is well printed, and cannot fail to render important service to the cause of sound charity.

---

THE annual report of the Liverpool Dispensaries is satisfactory in so far as it is said to prove the better health of Liverpool generally. The Mayor, in moving the adoption of the report and financial statement, said it was quite true that the death-rate of Liverpool had improved, thanks to the very good sanitary work which had been going on for some years, and it was to be hoped that the death-rate, which had been gradually declining, might continue to proceed in the same direction, if only because it meant less work for that society. Over 64,000 persons were treated last year by the dispensaries.

---

THE twenty-first annual meeting of the Governors of the National Hospital for Consumption, at Ventnor, was held at the London office, 34, Craven Street, Charing Cross, on February 25th. Mr. Herbert C. Saunders, Q.C., Chairman of the Board of Management, presided, in the unavoidable absence of the President, the Earl of Rosebery, who was presiding at the London County Council meeting. The number of in-patients under treatment had increased to 759, as compared with 735 in the previous year, and resided in nearly every county in the kingdom. The receipts reported amounted to £12,720, which included £3,795 from legacies. In addition to the ordinary expenses of maintenance (£9,350), the sum of £1,893 had been expended in improvements to the heating and ventilation. Regret was expressed at the loss of Mrs. Hamilton, a benefactress who used to defray the chaplain's stipend, which will now fall upon the general hospital funds. The retiring members of the Board were re-elected, and votes of thanks to the honorary and medical officers and the Chairman brought the proceedings to a close.

---

THE eighth annual report of the Hampstead Home Hospital and Nursing Institute should be read by all who are interested in the Pay Hospital system. It is a plain, business-like statement, full of instructive figures, and most encouraging to those who believe that every class of the population in this country would prefer to pay something, however small, for medical relief if a reasonable opportunity was everywhere afforded them to do so. Although the voluntary income—*i.e.*, the money given to the institute—is only half as much as it was four years ago—being £386 now, compared with £766 in 1886—the patients' payments and nurses' earnings have considerably more than doubled, amounting to £1,117 in 1889, as compared with £542 in 1886. The staff of nurses now consists of 20, and the accommodation at the hospital has been enlarged by the addition of a children's ward, containing 7 beds. We are glad to see that the committee have had the courage to resist the proposal to establish an out-patient department. Great credit is due to the Sister-Superintendent, whose name is unfortunately not given in the report, and to the staff, and we hope the festival dinner on the 20th inst. will produce at least £1,000. Mr. R. A. Owthwaite, who has acted as hon. secretary from the commencement, deserves warm commendation for the success of this effort, which is worthy of imitation, and the thanks of the public are due to him for the great amount of time and attention he has voluntarily given to this excellent and useful work.

